# Application and insights of targeted next-generation sequencing in a large cohort of 46,XY disorders of sex development in Chinese

**DOI:** 10.1186/s13293-024-00648-6

**Published:** 2024-09-16

**Authors:** Hongyu Chen, Guangjie Chen, Fengxia Li, Yong Huang, Linfeng Zhu, Yijun Zhao, Ziyi Jiang, Xiang Yan, Lan Yu

**Affiliations:** 1 Children’s Hospital, Zhejiang University School of Medicine, National Clinical Research Center for Child Health, Hangzhou, 310052 China; 2 Department of Urology, Children’s Hospital, Zhejiang University School of Medicine, National Clinical Research Center for Child Health, Hangzhou, 310052 China

**Keywords:** 46,XY disorders of sex development, Targeted next generation sequencing, Oligogenic variants, Genetic diagnostic rate

## Abstract

**Purpose:**

46,XY disorders of sex development (46,XY DSD) are characterized by incomplete masculinization of genitalia with reduced androgenization. Accurate clinical management remains challenging, especially based solely on physical examination. Targeted next-generation sequencing (NGS) with known pathogenic genes provides a powerful tool for diagnosis efficiency. This study aims to identify the prevalent genetic variants by targeted NGS technology and investigate the diagnostic rate in a large cohort of 46,XY DSD patients, with most of them presenting atypical phenotypes.

**Methods:**

Two different DSD panels were developed for sequencing purposes, targeting a cohort of 402 patients diagnosed with 46,XY DSD, who were recruited from the Department of Urology at Children’s Hospital, Zhejiang University School of Medicine (Hangzhou, China). The detailed clinical characteristics were evaluated, and peripheral blood was collected for targeted panels to find the patients’ variants. The clinical significance of these variants was annotated according to American College of Medical Genetics and Genomics (ACMG) guidelines.

**Results:**

A total of 108 variants across 42 genes were found in 107 patients, including 46 pathogenic or likely pathogenic variants, with 45.7%(21/46) being novel. Among these genes, *SRD5A2*, *AR*, *FGFR1*, *LHCGR*, *NR5A1*, *CHD7* were the most frequently observed. Besides, we also detected some uncommon causative genes like *SOS1*, and *GNAS*. Oligogenic variants were also identified in 9 patients, including several combinations *PROKR2/FGFR1/CYP11B1*,* PROKR2/ATRX*,* PROKR2/AR*,* FGFR1/LHCGR/POR*,* FGFR1/NR5A1*,* GATA4/NR5A1*,* WNT4/AR*,* MAP3K1/FOXL2*,* WNT4/AR*,* and SOS1/FOXL2*.

**Conclusion:**

The overall genetic diagnostic rate was 11.2%(45/402), with an additional 15.4% (62/402) having variants of uncertain significance. Additionally, trio/duo patients had a higher genetic diagnostic rate (13.4%) compared to singletons (8.6%), with a higher proportion of singletons (15.1%) presenting variants of uncertain significance. In conclusion, targeted gene panels identified pathogenic variants in a Chinese 46,XY DSD cohort, expanding the genetic understanding and providing evidence for known pathogenic genes’ involvement.

**Supplementary Information:**

The online version contains supplementary material available at 10.1186/s13293-024-00648-6.

## Abstract (plain language summary)

46,XY disorders of sex development (46,XY DSD) are conditions where individuals don’t fully develop male genitalia due to reduced androgen hormones. Diagnosing these conditions based only on physical exams is difficult. This study used advanced genetic testing called targeted next-generation sequencing (NGS) to identify common genetic variations in a large group of 46,XY DSD patients, many of whom had unusual symptoms. We examined 402 patients with DSD and a 46,XY karyotype, focusing on 142 candidate genes related to sex development. We found genetic variations in 107 patients, including 45 that were likely responsible for their condition. Some of these variations were new discoveries. The most commonly affected genes were *SRD5A2*, *AR*, *FGFR1*, *LHCGR*, *NR5A1*,* CHD7*. We also found that some patients had variations in multiple genes, suggesting complex genetic causes. Overall, we were able to diagnose 11.2% of patients based on our genetic testing, with another15.4% having uncertain results. Patients tested as a trio or duo (with their parents) had a higher diagnosis rate than those tested alone. This study helps expand our understanding of the genetic factors behind 46,XY DSD in the Chinese population.

**Supplementary Information:**

The online version contains supplementary material available at 10.1186/s13293-024-00648-6.

## Highlights

Two gene panels were designed, comprising 142 and 271 candidate genes associated with sex development, to sequence 402 patients with 46,XY DSD.

A total of 108 variants across 42 genes were identified in 107 patients, with 46 classified as pathogenic or likely pathogenic, including several novel variants. *SRD5A2*, *AR*, *FGFR1*, *LHCGR*, *NR5A1*, *CHD7* were among the most frequently observed genes with variants.

The overall genetic diagnostic rate was 11.2%, with an additional 15.4% of patients having variants of uncertain significance, and oligogenic variants were detected in 9 patients, suggesting complex genetic interactions in 46,XY DSD.

Patients with trio/duo genetic information had a higher genetic diagnostic rate (13.4%) compared to singletons (8.6%), underscoring the importance of parental genetic data.

Phenotypic variability and potential genetic heterogeneity underscore the complexity of 46,XY DSD, highlighting the necessity for further research and multi-center collaboration.

**Supplementary Information:**

The online version contains supplementary material available at 10.1186/s13293-024-00648-6.

## Introduction

Disorders of sexual development (DSDs) are a diverse group of congenital complex conditions in which the development of chromosomal, gonadal, or anatomical sex is atypical [1]. The exact incidence of DSD is not clear; but when considering all congenital genital anomalies, it can be as high as 1 in 200 to 300 births [2]. Signs of DSD can manifest at birth with atypical external genital anatomy, in childhood with bilateral inguinal hernias, in puberty with atypical secondary sex characteristics, or in adulthood with infertility [3]. Individuals with DSD often experience challenges related to self-esteem and gender identity [4]. Additionally, there is a significantly higher risk of gonadal tumors in patients with DSD compared to the general population [5]. The distress associated with overall health concerns, as well as the shame and fear of social stigma for individuals and their families, often lead to social withdrawal and isolation, consequently bring a profound psychopathological burden on them. Therefore, early diagnosis and intervention are necessary for the medical management of DSD. However, due to the complexity and heterogeneity of these conditions, clinical management of DSD can be challenging, interdisciplinary teams are needed.

According to the DSD international consensus, DSDs are classified into 46,XY, 46,XX, and sex chromosome DSD [1]. Among them, 46,XY DSD is the most common and complex type. It is characterized by female or ambiguous external genitalia such as hypospadias, cryptorchidism, micropenis, and testicular/gonadal dysgenesis, resulting from incomplete virilization with or without the presence of Mullerian structures [6]. Considering the aetio-pathogenesis of process of fetal sex differentiation, 46,XY DSD may be endocrine-related or not [7]. Non-endocrine disorders, such as isolated hypospadias, can arise from abnormal morphogenesis of testicular primordia [7]. On the other hand, endocrine-related 46,XY DSD can be attributed to early-onset gonadal failure, specifically affecting Leydig or Sertoli cell functions, or defects in male hormone at target tissues during embryogenesis, puberty, and adulthood [3]. Impaired secretion of anti-mullerian hormone (AMH) from Stertoli cells will lead to the failure of Mullerian ducts regression. Similarly, decreased androgen production such as testosterone (T) or dihydrotestosterone (DHT) from Leydig cells can impact the development of Wolffian ducts, influencing the formation of epidymides, vasa deferentia, and seminal vesicles [8].

Mutational and functional analyses of patients with DSDs and mouse models have revealed a large number of genes involved in male differentiation [9].These genes can be categorized according to their functions as follows: (1) genes related to the development and diferentiation of gonadal, such as *SRY*, *NR5A1*, *WT1*, *MAP3K1*, *GATA4*, and *MAMLD1*. (2) genes associated with hormone synthesis and action, including steroid hormone and androgen, such as *AR*, *SRD5A2*, *AKR1C2*, *CHD7*, *LHCGR*, *ANOS1*, and *CYP11A1*. In addition to single gene mutations, copy number variations (CNVs) have also been shown to be associated with DSD. For instance, deletions of 9p24.3 and 10q26.1, as well as duplications of Xp21.2 and 1p35, have been identified [10].

The clinical phenotype of 46,XY DSD patients often presents similar manifestations, making accurate diagnose challenging based solely on physical examination. However, having knowledge of the pathogenic genes involved in these patients can enhance diagnosis efficiency, potentially reducing the need for additional expensive biochemical and radiological assessments. The decreasing costs of Targeted Next-Generation Sequencing (NGS) have rendered this approach increasingly viable in clinical practice. The primary objective of this study was to identify gene variants in a large cohort of 46,XY DSD patients and specifically investigate any unique gene variants or hotspots within the Chinese population, and reveal the prevalent variants of 46,XY DSD by comparing with previous studies. By doing so, we aimed to contribute substantial evidence to facilitate early diagnosis and intervention for affected individuals.

## Methods

### Patient recruitment and clinical assessment

We conducted a retrospective analysis involving 402 patients suspected of DSD at the Department of Urology, Children’s Hospital, Zhejiang University School of Medicine, between 1/2017 and 12/2020, for further investigation. All patients were subjected to detailed clinical and genital examinations, family history pedigree analysis, and the assessment of associated abnormalities. The patient inclusion criteria were as follows: (1) patients with 46,XY karyotype and (2) patients with external genital malformation or gonadal dysplasia, including female external genitalia, clitoromegaly, ambiguous external genitalia, perineal hypospadias, cryptorchidism, and micropenis.

The study was ethically approved by the Human Subjects Committees of Children’s Hospital, Zhejiang University School of Medicine(2018-IRB-076). All procedures performed in studies involving human participants were in accordance with the ethical standards of the Ethical Committee. An informed consent was obtained from the patients or their guardians. Written informed consent was obtained from the patients, the patients’ parents, or their legal guardians.

### Targeted gene panel

Two gene panels were designed for screening 46,XY DSD, comprising 142 candidate genes (M014) and 271 genes (KY043), respectively. Genes included in these panels were selected based on the knowledge of DSD sourced from PubMed, OMIM, and other genetic testing registry databases. These genes were classified into several categories, including synthesis or action of androgen, development and differentiation of gonadal, synthesis and activation of Steroid hormone, syndromic disorders, development and differentiation of germ cell, and others) (Supplementary Tables 3 & 4).

### DNA library preparation

Genomic DNA was extracted from peripheral blood leukocytes using the Qiagen DNA Blood kit (Qiagen, Dusseldorf, Germany) from each sample. A minimum of 3 µg DNA was fragmented to an average size of 180 bp using a Bioruptor sonicator (Diagenode). Paired-end sequencing libraries were then prepared using a DNA sample prep reagent set 1 (NEBNext). The library preparation was followed by the recommended protocols from Illumina, the process involved end repair, adapter ligation, and polymerase chain reaction (PCR) amplication.

### Targeted genes enrichment and sequencing

The exon regions and exon-intron boundaries of the target genes were captured using GenCap customized DSD Kit (MyGenostics Inc. Beijing, China) according to previously described methods [11]. In brief, 1 µg DNA library was mixed with Buffer BL and GenCap DSD probe (MyGenostics, Beijing, China). The mixture was then heated at 95 °C for 7 min, followed by 65 °C for 2 min in a PCR machine. Subsequently, 23 µl of the 65 °C pre-warmed Buffer HY (MyGenostics Inc, Beijing, China) was added, and the mixture was maintained at 65 °C with the PCR lid heat on for 22 h for hybridization. After that, 50 µl MyOne beads (Life Technology) underwent washing in 500µL 1X binding buffer three times and were then resuspended in 80 µl 1X binding buffer. Following this, 64 µl of 2X binding buffer was added to the hybrid mixture, and the entire mixture was transferred into a tube containing 80 µl MyOne beads. The mixure was rotated for 1 h on a rotator. The beads were then washed with WB1 buffer at room temperature for 15 min once and with WB3 buffer at 65 °C for 15 min three times. Afterward, the bound DNA was eluted with Buffer Elute. The eluted DNA was finally amplified for 15 cycles using the following program: 98 °C for 30 s (1 cycle); 98 °C for 25 s, 65 °C for 30 s, 72 °C for 30 s (15 cycles); 72 °C for 5 min (1 cycle). The PCR product was purified using SPRI beads (Beckman Coulter) according to manufacturer’s protocol. The enriched libraries were sequenced on Illumina HiSeq X ten sequencer with paired read of 150 bp.

### Variant calling and annotation

Paired-end sequence reads in fastq files were filtered to remove low-quality reads by using Fastp [12] with default parameters. After ensuring quality control, the clean reads were aligned to the UCSC hg19 human reference genome with Burrows-Wheeler Aligner Maximal Exact Match (BWA-MEM) [13]. Duplicated reads were marked with Picard tools [14]. Variants of Single Nucleotide Variations (SNVs) and Insertions/deletions (Indels) were called using Genome Analysis Toolkit (GATK 4.1.7.0) [15] HaplotypeCaller to generate gVCF files for joint genotyping. All samples were jointly genotyped and variants were selected with GATK Selectvariants for hard filtering with the following criteria; (a) Quality by depth < 2; (b) Mapping Quality < 40; (c) approximate read depth < 6; (d) phred-scaled p-value using Fisher’s exact test to detect strand bias (FS) > 60 for SNVs and > 200 for Indels. ANNOVAR [16] was used to annotate variant function, in silico predictions of deleteriousness, and variant populations frequencies.

### Pathogenicity analysis of the variants

Variants were classified as pathogenic (P), likely pathogenic (LP), variant of uncertain significance (VUS), likely benign, or benign according to the American College of Medical Genetics and genomics guidelines [17]. Pathogenic and likely pathogenic variants were prioritized if indicated in Clinvar, Human Gene Mutation Database (HGMD, 2022.12), or had been reported to be associated with DSD. The remaining variants were filtered and prioritized based on MAF less than 0.1% in all population datasets, and function annotation of missense, inframe, frameshift indels, canonical splice site, or nonsense variants. The pathogenicity of variants was further calibrated using Varsome [18]. Variants identified as pathogenic, likely pathogenic, or VUS were verified by Sanger sequencing and further confirmation of origin with available parental samples, benign and likely benign variants were discarded.

## Results

### Sequencing quality and cohort characteristics

The Average sequencing depth on target for panel M014 and KY043 is 265× (range from 127× to 660×) and 461× (range from 165× to 1316×), respectively (Supplementary Fig. 1A). More than 76% of targeted regions were covered with greater than 20× for each sample in M014 panel, while more than 90% of targeted regions were covered with greater than 20× for samples in KY043 panel (Supplementary Fig. 1B).

Total of 402 patients with a 46,XY karyotype and genetic testing result have been recruited for analysis. Among them, 185 are singletons, 30 are duo samples, and 187 are trio-based samples. The mean age at diagnosis was 3.4 ± 3 years old, specifically, 54 patients were first assessed at minipubertal age (< 6 months), 294 patients at prepubertal age (6 months-9 years), 54 patients at pubertal age (9–18 years). Majority of patients are Han Chinese, and 7 of them are minority Chinese.

As listed in Table 1, the patients presented with a variety of DSD phenotypes. Seventeen patients have a determined etiologic diagnosis. Among the remaining patients with unknown etiology, the most common clinical manifestation was the micropenis and hypospadias, accounted for 50.6% (205/402) and 22.5% (91/402), respectively. Three of those 402 patients were raised as females because of ambiguous genitalia and need for male sex assignment after genetic diagnosis and karyotype (supplementary Table 1).

**Table 1:** Clinical characteristics of patients in our cohort

	Patients, *n*(%)	Trios/duos, *n*	Singletons, *n*
**Age**	3.5 ± 3(Years)		
Minipubertal age (< 6 months)	54	26	28
Prepubertal age (6 months-9 years)	294	164	130
Pubertal age (9–18 years)	54	27	27
total	402	217	185
**Ethnicity**			
Han	395(98.3%)		
Others	7(1.7%)		
**Clinical diagnosis**			
Gonadal dysgenesis(GD)	10(2.5%)	2	8
5α-reductase 2 defciency	6(1.5%)	5	1
Androgen insensitivity(AIS)	3(0.7%)	0	3
**46 XY**,** DSD of clinically unknown etiology**			
Hypospadias	91(22.5%)	52	39
Hypospadias, Micropenis	4(1.2%)	1	3
Hypospadias, Cryptorchidism	6(1.5%)	1	5
Cryptorchidism(bilateral)	12(2.9%)	6	6
Cryptorchidism(right or left )	20(5.1%)	10	10
Cryptorchidism, Micropenis	12(2.9%)	6	6
Micropenis	205(50.6%)	120	85
Ambiguous genitalia	2(0.5%)	0	2
Ambiguous genitalia, Hypospadias	5(1.5%)	2	3
Hypospadias, gonadal dysgenesis	3(0.7%)	0	3
CHD with DSD-related phenotype	10(2.4%)	7	3
Growth retardation with DSD-related phenotype	3(0.7%)	1	2
Hypospadias with other malformations	6(1.5%)	2	4
Micropenis with other malformations	3(0.7%)	2	1
Cryptorchidism with other malformations	1(0.2%)	0	1
total	402	217	185

*Note* The diagnostic rate for each phenotype was calculated by dividing the number of positive samples by the total number of patients with that phenotype

### Variants analysis

**Fig. 1:**
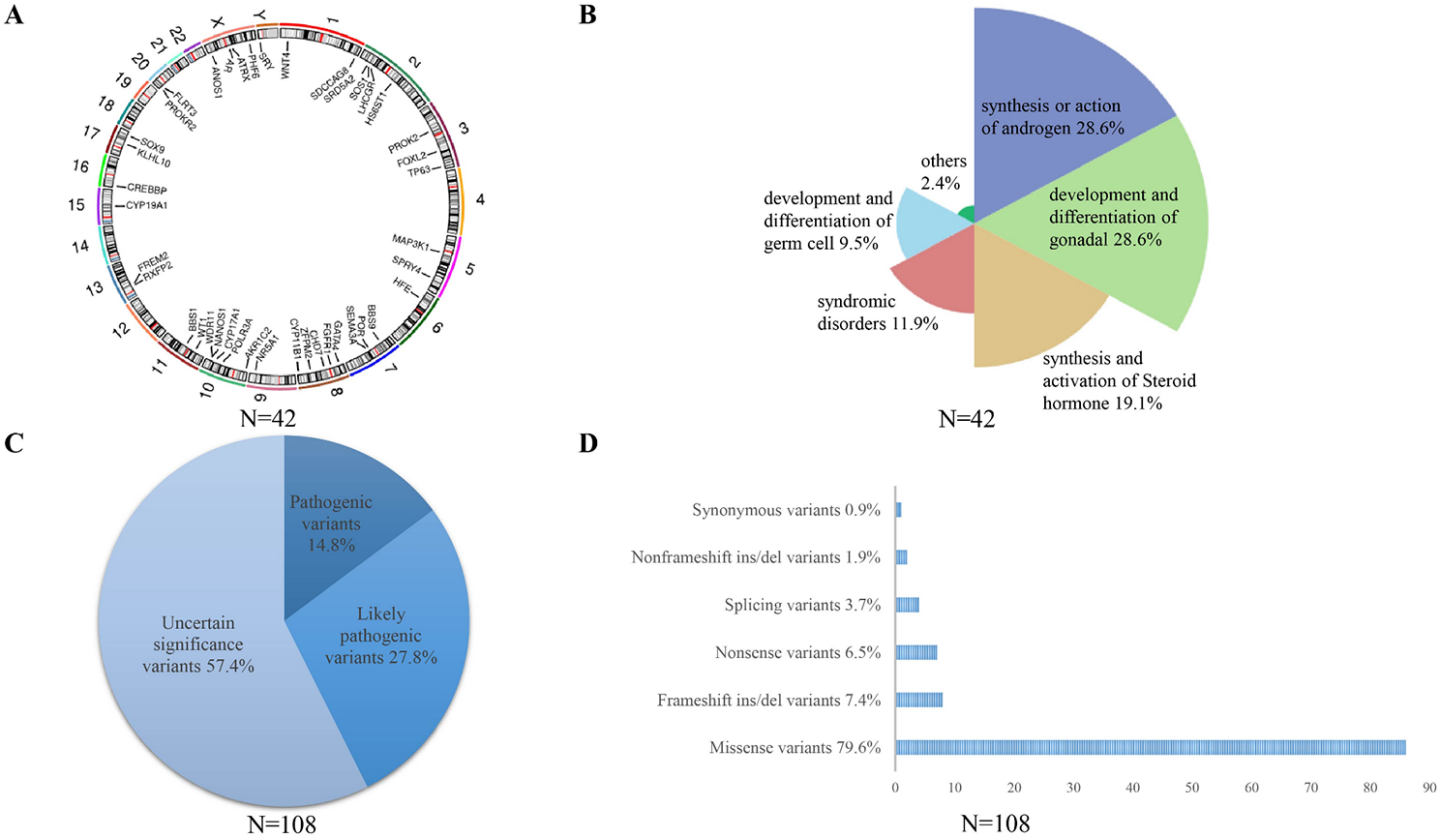
Genetic diagnosis of the 46,XY DSD cohort. (**A**) Location of identified genes on chromosomes; (**B**) Proportion of different categories of DSD genes; (**C**) Proportion of variants at different evidence levels; (**D**) Proportion of different variants type

After filtering by the ACMG guideline, a total of 108 variants involved 42 genes were positively detected in 107 patients (Supplementary Table 1). These genes were distributed all around the chromosomes (Fig. 1A). Furthermore, variants were mainly enriched in genes associated with the synthesis or action of androgen (28.6%), development and differentiation of gonadal (28.6%), and synthesis and activation of steroid hormone (19.1%) (Fig. 1B). For the 108 variants, 14.8% (16/108) were pathogenic variants, 27.8% (30/108) were likely pathogenic variants, and the remaining 57.4% (62/108) were VUS (Fig. 1C). Among these variants, missense variants were the most common type accounting for 79.6% (86/108), followed by frameshift variants accounting for 7.4% (8/108), and nonsense variants for 6.5% (7/108) (Fig. 1D). Additionally, four splicing variants and two nonframeshift insertion/deletion variants were identified. One patient had a VUS synonymous variant compound with a pathogenic missense variant in *SRD5A2* gene (Fig. 1D, supplementary Table 1).

**Fig. 2:**
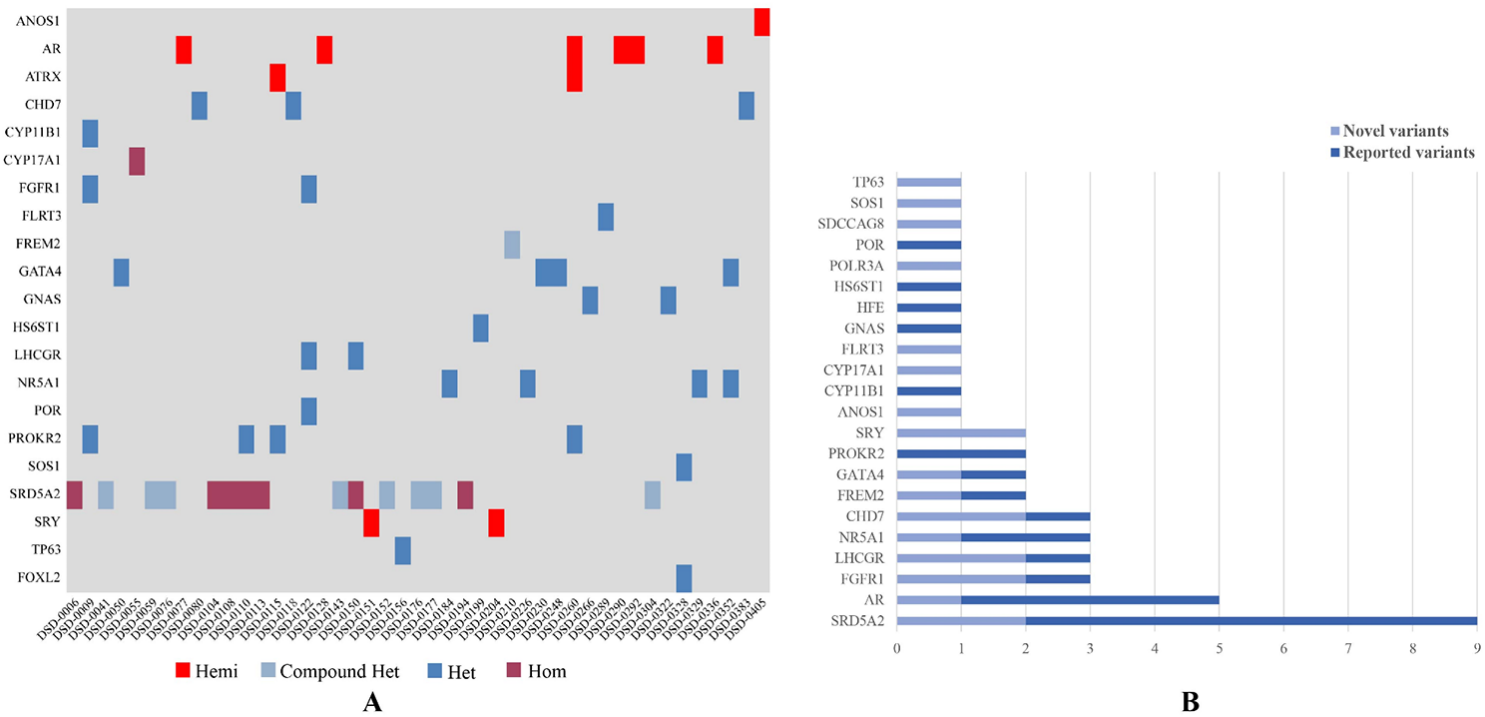
Distribution of the P/LP variants identified in the 46,XY DSD cohort. (**A**) Variants identified in patients with the definitive genetic diagnosis; (**B**) Variants identified in 22 genes in this study

The clinical and molecular characteristics including main identified variants, clinical features, karyotypes, and transmission of positive cases are summarized in Table 1. And we visualized patients- genes information in patients in Fig. 2A. Information of the negative cases and the details of the evidence level applied appropriately are provided in Supplementary Table S2. *SRD5A2* was the most frequent gene, including 2 novel variants and 7 reported variants (Fig. 2B) occurring in 15 patients (Table 2).

**Table 2:** Clinical and molecular characteristics of the 45 positive cases in our cohort

						Amino acid change							
Gene	Patient’s ID	Phenotype	Inheritance	Transcript	exon	Nucleotide Change	Amino acid change	Frequency(ExAC_EAS)	Heterozygosity	Transmission	Variant type	Pathogenicity	Reference(PMID or clinvarID)
SRD5A2	**DSD-0006**	ambiguous genitalia, hypospadias, Multiple malformations	AR	NM_000348	exon4	c.G607A	p.G203S	0.0012	Hom	F/M	Missense	P	36,016,984
PROKR2	**DSD-0009**	Micropenis, Cryptorchidism(bilateral)	AD	NM_144773	exon3	c.G491A	p.R164Q	.	Het	De novo	Missense	P	18,826,963
FGFR1	**DSD-0009**	Micropenis, Cryptorchidism(bilateral)	AD	NM_023110	exon13	c.G1664-1T		.	Het	De novo	Splicing	P	novel
CYP11B1	**DSD-0009**	Micropenis, Cryptorchidism(bilateral)	AR	NM_000497	exon4	c.G779A	p.W260X	.	Het	De novo	Nonsense	LP	28,962,970
SRD5A2	**DSD-0041**	5α-reductase 2 defciency, hypospadias	AR	NM_000348	exon5	c.G680A	p.R227Q	0.0056	Het	Mother	Missense	LP	31,186,340
SRD5A2	**DSD-0041**	5α-reductase 2 defciency, hypospadias	AR	NM_000348	exon1	c.T59C	p.L20P	0.0001	Het	Father	Missense	P	18,314,109
GATA4	DSD-0050	Micropenis	AD	NM_001308093	exon1	c.C487T	p.P163S	0	Het	Mother	Missense	LP	31,513,339
CYP17A1	DSD-0055	gonadal dysgenesis(complet)	AR	NM_000102	exon5	c.A968G	p.Q323R	.	Hom	F/M	Missense	LP	novel
SRD5A2	**DSD-0059**	Micropenis	AR	NM_000348	exon5	c.G680A	p.R227Q	0.0056	Het	Father	Missense	LP	31,186,340
SRD5A2	**DSD-0059**	Micropenis	AR	NM_000348.4	exon1	c.G196A	p.G66R	0.0023	Het	Mother	Missense	VUS	31,219,235
SRD5A2	**DSD-0076**	hypospadias	AR	NM_000348	exon6	c.G737A	p.R246Q	0.0009	Het	Father	Missense	LP	32,346,305
SRD5A2	**DSD-0076**	hypospadias	AR	NM_000348	exon5	c.G680A	p.R227Q	0.0056	Het	Mother	Missense	LP	31,186,340
AR	DSD-0077	ambiguous genitalia	XLR	NM_000044	exon8	c.C2656T	p.H886Y	.	Hemi	Mother	Missense	LP	VCV002941472.1
CHD7	DSD-0080	gonadal dysgenesis(complet)	AD	NM_017780	exon3	c.1969dupA	p.E658Rfs*18	.	Het	De novo	frameshift insertion	P	novel
SRD5A2	DSD-0104	5α-reductase 2 defciency, hypospadias, micropenis	AR	NM_000348	exon5	c.G680A	p.R227Q	0.0056	Hom	F/M	Missense	LP	31,186,340
SRD5A2	DSD-0108	hypospadias	AR	NM_000348	exon5	c.G680A	p.R227Q	0.0056	Hom	F/M	Missense	LP	31,186,340
PROKR2	**DSD-0110**	Micropenis	AD	NM_144773	exon3	c.G533C	p.W178S	0.0023	Het	unknown	Missense	LP	36,317,218
SRD5A2	**DSD-0110**	Micropenis	AR	NM_000348	exon5	c.G680A	p.R227Q	0.0056	Hom	F/M	Missense	LP	31,186,340
SRD5A2	DSD-0113	5α-reductase 2 defciency	AR	NM_000348	exon5	c.G680A	p.R227Q	0.0056	Hom	F/M	Missense	LP	31,186,340
PROKR2	**DSD-0115**	Micropenis, Cryptorchidism(right), inguinal hernia	AD	NM_144773	exon3	c.G533C	p.W178S	0.0023	Het	De novo	Missense	P	36,317,218
ATRX	**DSD-0115**	Micropenis, Cryptorchidism(right), inguinal hernia	XLR	NM_138270	exon16	c.C4643T	p.P1548L	.	Hemi	unknown	Missense	VUS	novel
CHD7	DSD-0118	Micropenis, Cryptorchidism(bilateral)	AD	NM_017780	exon12	c.3009_3010insATT	p.I1004_T1005insI	.	Het	De novo	frameshift insertion	LP	novel
FGFR1	**DSD-0122**	Micropenis	AD	NM_001174063	exon3	c.246_247del	p.E84Gfs*26	.	Het	De novo	frameshift deletion	P	novel
LHCGR	**DSD-0122**	Micropenis	AR	NM_000233	exon1	c.135delC	p.G46Afs*18	.	Het	De novo	frameshift deletion	LP	novel
POR	**DSD-0122**	Micropenis	AR	NM_000941	exon12	c.G1370A	p.R457H	0.001	Het	unknown	Missense	P	36,518,257
AR	DSD-0128	hypospadias	XLR	NM_000044	exon6	c.G2387T	p.G796V	.	Hemi	Mother	Missense	LP	novel
SRD5A2	**DSD-0143**	hypospadias	AR	NM_000348	exon6	c.G737A	p.R246Q	0.0009	Het	Mother	Missense	LP	32,346,305
SRD5A2	**DSD-0143**	hypospadias	AR	NM_000348	exon1	c.C16T	p.Q6X	0.0002	Het	Father	Nonsense	P	35,331,321
SRD5A2	DSD-0150	Micropenis	AR	NM_000348	exon5	c.G680A	p.R227Q	0.0056	Hom	F/M	Missense	LP	31,186,340
SRY	DSD-0151	gonadal dysgenesis, Micropenis, Cryptorchidism(bilateral)	YL	NM_003140	exon1	c.C236T	p.A79V	.	Hemi	De novo	Missense	P	novel
SRD5A2	**DSD-0152**	Micropenis	AR	NM_000348.4	exon4	c.418_419insC	p.Y140Sfs*7	.	Het	unknown	frameshift insertion	LP	novel
SRD5A2	**DSD-0152**	Micropenis	AR	NM_000348.4	exon4	c.G417T	p.W139C	0	Het	unknown	Missense	LP	novel
TP63	DSD-0156	hypospadias	AD	NM_001114981	exon11	c.C1378T	p.Q460X	.	Het	unknown	Nonsense	LP	novel
SRD5A2	**DSD-0176**	5α-reductase 2 defciency, hypospadias	AR	NM_000348	exon6	c.G737A	p.R246Q	0.0009	Het	Father	Missense	LP	32,346,305
SRD5A2	**DSD-0176**	5α-reductase 2 defciency, hypospadias	AR	NM_000348	exon1	c.T59C	p.L20P	0.0001	Het	Mother	Missense	P	18,314,109
SRD5A2	**DSD-0177**	Micropenis	AR	NM_000348	exon5	c.G680A	p.R227Q	0.0056	Het	unknown	Missense	LP	31,186,340
SRD5A2	**DSD-0177**	Micropenis	AR	NM_000348.4	exon1	c.C81T	p.Val27=	.	Het	unknown	Synonymous	VUS	novel
NR5A1	DSD-0184	hypospadias	AD	NM_004959	exon4	c.G251A	p.R84H	0	Het	Father	Missense	P	30,425,642
SRD5A2	DSD-0194	Micropenis	AR	NM_000384	exon6	c.C702G	p.F234L	0.0128	Hom	F/M	Missense	P	31,031,332
HS6ST1	DSD-0199	Micropenis	AD	NM_004807	exon2	c.C553T	p.R185X	0.0004	Het	unknown	Nonsense	LP	31,371,345
SRY	DSD-0204	hypospadias, inguinal hernia	YL	NM_003140	exon1	c.G448T	p.A150S	.	Hemi	De novo	Missense	LP	novel
FREM2	**DSD-0210**	hypospadias	AR	NM_207361	exon1	c.C1603T	p.R535C	0.0016	Het	Mother	Missense	LP	VCV000311949.12
FREM2	**DSD-0210**	hypospadias	AR	NM_207361	exon1	c.C3014T	p.T1005I	0.0005	Het	Father	Missense	LP	novel
NR5A1	DSD-0226	hypospadias	AD	NM_004959	exon7	c.T1210G	p.Y404D	.	Het	De novo	Missense	P	VCV000216976.1
GATA4	DSD-0230	gonadal dysgenesis, CHD(Atrial septal defect)	AD	NM_001308093	exon1	c.C487T	p.P163S	0	Het	Mother	Missense	LP	31,513,339
GATA4	DSD-0248	hypospadias	AD	NM_001308093	exon1	c.C487T	p.P163S	0	Het	De novo	Missense	LP	31,513,339
PROKR2	**DSD-0260**	Micropenis	AD	NM_144773	exon3	c.G533C	p.W178S	0.0023	Het	De novo	Missense	P	36,317,218
AR	**DSD-0260**	Micropenis	XLR	NM_000044	exon1	c.A179T	p.Q60L	.	Het	Mother	Missense	VUS	26,804,919
ATRX	**DSD-0260**	Micropenis	XLR	NM_138270	exon8	c.A2352C	p.E784D	0.0002	Hemi	Mother	Missense	VUS	VCV002191696.1
GNAS	DSD-0266	Micropenis	AD	NM_080425	exon1	c.G424T	p.G142X	.	Het	unknown	Nonsense	LP	32,157,680
FLRT3	DSD-0289	Micropenis	AD	NM_013281	exon2	c.78_79insC	p.M27Hfs*3	.	Het	Mother	frameshift insertion	LP	novel
AR	DSD-0290	hypospadias	XLR	NM_000044	exon4	c.A1976G	p.K659R	.	Hemi	Mother	Missense	LP	VCV002445341.2
AR	DSD-0292	hypospadias	XLR	NM_000044	exon8	c.A2659G	p.M887V	0.0008	Hemi	unknown	Missense	LP	28,261,839
SRD5A2	**DSD-0304**	5α-reductase 2 defciency, hypospadias, micropenis	AR	NM_000348	exon5	c.G680A	p.R227Q	0.0056	Het	Mother	Missense	LP	31,186,340
SRD5A2	**DSD-0304**	5α-reductase 2 defciency, hypospadias, micropenis	AR	NM_000348.4	exon5	c.656del	p.F219fs	0.0001	Het	Father	frameshift deletion	P	37,147,882
GNAS	DSD-0322	Micropenis	AD	NM_080425	exon1	c.G424T	p.G142X	.	Het	unknown	Nonsense	LP	32,157,680
SOS1	**DSD-0328**	hypospadias	AD	NM_005633	exon10	c.C1410A	p.C470X	.	Het	unknown	Nonsense	LP	novel
FOXL2	**DSD-0328**	hypospadias	AD	NM_023067	exon1	c.A737G	p.K246R	.	Het	unknown	Missense	VUS	32,332,759
NR5A1	DSD-0329	Cryptorchidism(right)	AD	NM_004959	exon4	c.G842A	p.R281H	.	Het	unknown	Missense	LP	novel
AR	DSD-0336	hypospadias	XLR	NM_000044	exon5	c.G2191A	p.V731M	0.0006	Hemi	Mother	Missense	LP	35,924,163
GATA4	**DSD-0352**	Micropenis, Cryptorchidism(bilateral)	AD	NM_001308093	exon1	c.35_36insGCC	p.P15_G16insP	.	Het	unknown	frameshift insertion	LP	novel
NR5A1	**DSD-0352**	Micropenis, Cryptorchidism(bilateral)	AD	NM_004959	exon7	c.G1333A	p.E445K	.	Het	unknown	Missense	VUS	novel
CHD7	DSD-0383	Cryptorchidism(right), micropenis	AD	NM_017780	exon22	c.G5050A	p.G1684S	.	Het	De novo	Missense	P	21,158,681
ANOS1	DSD-0405	gonadal dysgenesis (complet)	XLR	NM_000216	exon13	c.1877_1887del	p.P626Lfs*37	.	Hemi	unknown	frameshift deletion	LP	novel

### Oligogenic variants

In addition to monogenic variants, oligogenic variants were also identified in several patients. After excluding the variants without interaction or combinations with low pathogenicity by ORVAL(Oligogenic Resource for Variant AnaLysis (ibsquare.be), we finally identified 7 patients with digenic variants and 2 patients with trigenic variants, involving various gene combinations and including *PROKR2/FGFR1/CYP11B1*, *PROKR2/ATRX*, *PROKR2/AR*, *FGFR1/LHCGR/POR*, *FGFR1/NR5A1*, *GATA4/NR5A1*, *WNT4/AR*, *MAP3K1/FOXL2*, and *SOS1/FOXL2* (Table 3). All 9 patients presented with phenotypes of micropenis, cryptorchidism, or hypospadias.

**Table 3:** Oligogenic variants in our cohort

Patient’s ID	Phenotype	Number of genes	Number of variants	Gene	Gene category	Transcript	Nucleotide change	Amino acid change	Variant type	Heterozygosity	Pathogenicity	Inheritance	Reference(PMID)	ORVAL prediction		
														Combination	VarCoPP score	Predicted pathogenicity
DSD-0009	Micropenis, Cryptorchidism(bilateral)	3	3	PROKR2	synthesis or action of androgen	NM_144773	c.G491A	p.R164Q	Missense	Het	P	De novo	18,826,963	FGFR1/ PROKR2	0.98	Disease-causing with 99.9% confidence
DSD-0009				FGFR1	synthesis or action of androgen	NM_023110	c.1664-1G > T		Splicing	Het	LP	De novo	novel	FGFR1/CYP11B1	0.835	Disease-causing with 99% confidence
DSD-0009				CYP11B1	synthesis and activation of Steroid hormone	NM_000497	c.G779A	p.W260X	Nonsense	Het	P	De novo	28,962,970	CYP11B1/PROKR2	0.7975	Disease-causing with 99% confidence
DSD-0115	Micropenis, Cryptorchidism(right), inguinal hernia	2	2	PROKR2	synthesis or action of androgen	NM_144773	c.G533C	p.W178S	Missense	Het	P	De novo	36,317,218		0.7425	Candidate disease-causing
DSD-0115				ATRX	Syndromic disorders	NM_138270	c.C4643T	p.P1548L	Missense	Hemi	VUS	unknown	novel			
DSD-0260	Micropenis	2	2	PROKR2	synthesis or action of androgen	NM_144773	c.G533C	p.W178S	Missense	Het	P	De novo	36,317,218	PROKR2/AR	0.92	Disease-causing with 99% confidence
DSD-0260				AR	synthesis and activation of Steroid hormone	NM_000044	c.A179T	p.Q60L	Missense	Hemi	VUS	Mother	26,804,919			
DSD-0260				ATRX	synthesis and activation of Steroid hormone	NM_138270	c.A2352C	p.E784D	Missense	Hemi	VUS	Mother	VCV002191696.1			
DSD-0122	Micropenis	3	3	FGFR1	synthesis or action of androgen	NM_001174063	c.246_247del	p.E84Gfs*26	frameshift deletion	Het	P	De novo	novel	FGFR1/LHCGR	NA	No combination
DSD-0122				LHCGR	synthesis and activation of Steroid hormone	NM_000233	c.135delC	p.G46Afs*18	frameshift deletion	Het	VUS	De novo	novel	LHCGR/POR	NA	No combination
DSD-0122				POR	synthesis and activation of Steroid hormone	NM_000941	c.G1370A	p.R457H	Missense	Het	VUS	unknown	36,518,257	FGFR1/POR	0.9225	Disease-causing with 99.9% confidence
DSD-0088	hypospadias	2	2	FGFR1	synthesis or action of androgen	NM_001174063	c.T20G	p.L7R	Missense	Het	VUS	unknown	37,805,574		0.98	Disease-causing with 99.9% confidence
DSD-0088				NR5A1	development and differentiation of gonadal	NM_004959	c.C1019T	p.A340V	Missense	Het	VUS	unknown	30,668,521			
DSD-0352	Micropenis, Cryptorchidism(bilateral)	2	2	GATA4	development and differentiation of gonadal	NM_001308093	c.35_36insGCC	p.P15_G16insP	frameshift insertion	Het	LP	unknown	novel		0.9775	Disease-causing with 99.9% confidence
DSD-0352				NR5A1	development and differentiation of gonadal	NM_004959	c.G1333A	p.E445K	Missense	Het	VUS	unknown	novel			
DSD-0323	Micropenis	2	2	AR	synthesis and activation of Steroid hormone	NM_000044	c.C1219G	p.R407G	Missense	Hemi	LP	Mother	novel		0.9925	Disease-causing with 99.9% confidence
DSD-0323				WNT4	development and differentiation of gonadal	NM_030761	c.G847A	p.D283N	Missense	Het	VUS	unknown	novel			
DSD-0328	hypospadias	2	2	SOS1	Syndromic disorders	NM_005633	c.C1410A	p.C470X	Nonsense	Het	LP	unknown	novel		0.9125	Disease-causing with 99.9% confidence
DSD-0328				FOXL2	development and differentiation of gonadal	NM_023067	c.A737G	p.K246R	Missense	Het	VUS	unknown	32,332,759			
DSD-0058	Micropenis	2	2	MAP3K1	development and differentiation of gonadal	NM_005921	c.A1664G	p.D555G	Missense	Het	VUS	unknown	novel		0.9475	Disease-causing with 99.9% confidence
DSD-0058				FOXL2	development and differentiation of gonadal	NM_023067	c.G1036C	p.A346P	Missense	Het	VUS	unknown	novel			

### Molecular genetic diagnosis

**Fig. 3:**
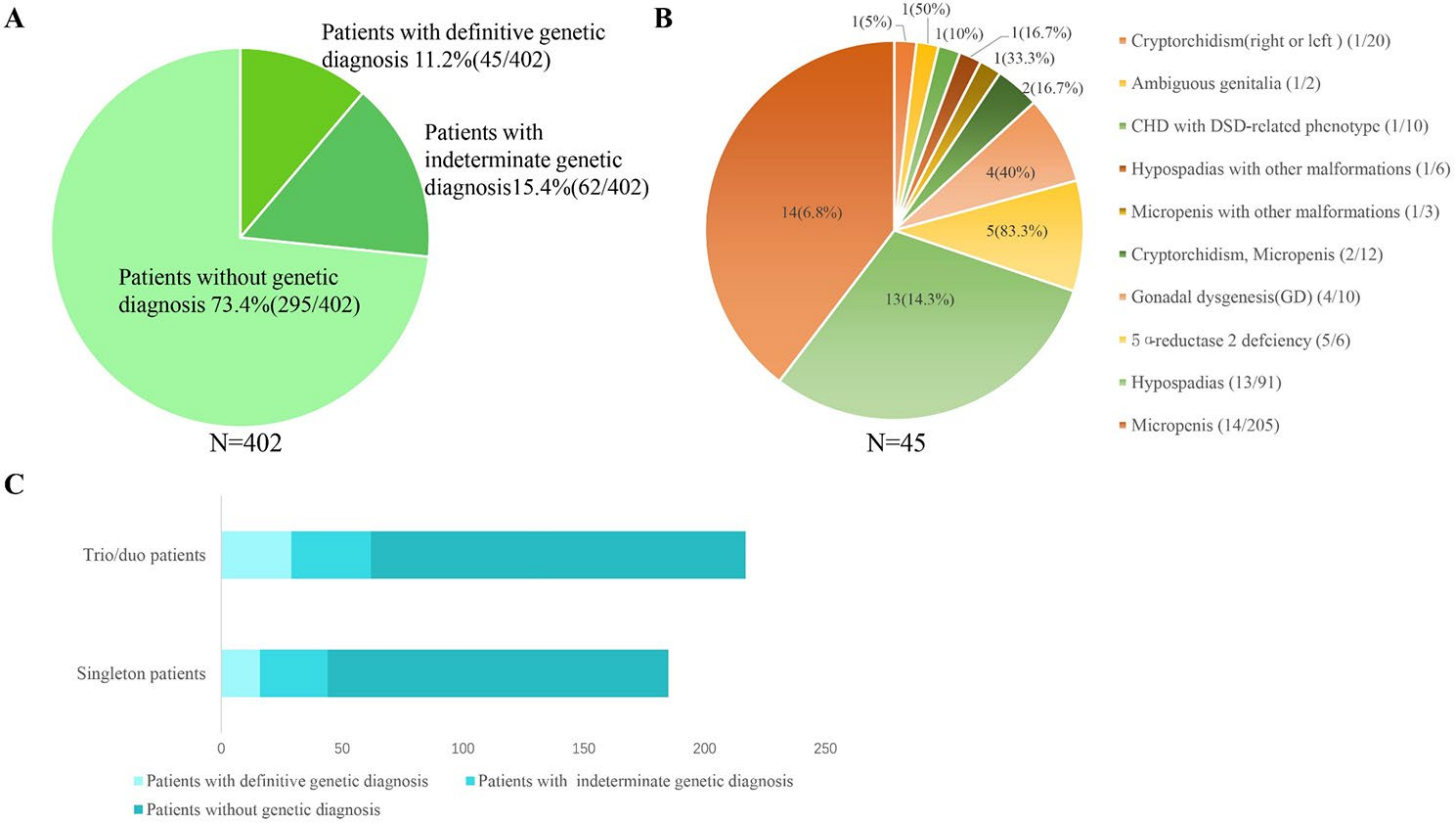
Features of the prevalent variants in 402 46,XY DSD patients. (**A**) a genetic diagnosis of the 402 patients; (**B**) diagnostic rate in patients with the different phenotype categorization; (**C**) the genetic diagnosis in singleton and trio/duo patients

An overall diagnostic rate was 11.2% (45/402) with pathogenic and likely pathogenic variants. There were another 15.4% of patients with VUS variants (Fig. 3A). Among those 45 positive cases, the genetic diagnostic rate was over 50% in patients who have determined etiologic diagnoses with 5α-reductase 2 deficiency and GD. For those with clinically unknown etiology, the number of patients with isolated phenotype of micropenis and hypospadias was 205 and 91, respectively; and the diagnostic rate was 6.8% (14/205) and 14.3% (13/91) (Fig. 3B). Among those trio/duo patients, 13.4% (29 of 217) of patients have been determined as genetic diagnostic patients with P/LP variants; while in singletons,8.6% (16 of 185) of patients were with genetic diagnostic findings. The proportion of VUS in singleton patients (28 of 185, 15.1%) was similar to that in trios (33 of 217, 15.2%) (Fig. 3C).

## Discussion

This study conducted a retrospective analysis in patients devolved to 46,XY DSD from 2017 to 2020, and two panels were designed for investigating genetic variants. In contrast to other research, our targeted panel encompasses a broader spectrum of candidate genes, including those crucial for sex development regulation. However, we have still missed some latest genes associated with DSD, which is a flaw in our design. As a result, 108 variants involved 42 genes were identified in 107 patients, with 46 of these variants being pathogenic or likely pathogenic. The overall diagnostic rate was 11.2% (45/402) and excluded the carriers pathogenic or likely pathogenic variants. Among these variants, *SRD5A2* and *AR* were the most frequently affected genes, followed by *FGFR1*, *LHCGR*, *NR5A1 and CHD7*. These findings are basically consistent with previous studies conducted in China [19–21].

Notably, in our study, a majority of patients exhibited various DSD-related phenotype without a clear etiology. This observation is likely attributed to the fact that many patients opt for genetic diagnosis only when the cause of the disease is not readily apparent. However, obtaining a genetic diagnosis is of importance, not only for elucidating the etiology but also for guiding patient management, including considerations related to potential gender development, assessment of adrenal and gonadal function, and evaluating the risk of sexual adenocarcinoma, associated morbidity, and long-term outcomes [22, 23]. Previous research has indicated that the risk of germ cell tumors in patients with gonadal dysgenesis can reach up to 30%, while in AIS patients, it is approximately 15% [24]. Therefore, it is important for 46,XY DSD patients to identify the etiology as early as possible.

5α-reductase 2 deficiency is an autosomal recessive inherited disease caused by homozygous or compound heterozygous variants of *SRD5A2* gene. In our study, we identified 12 different *SRD5A2* variants in 17 unrelated 46,XY DSD patients. Of these, only three variants were novel, one of which was a synonymous variant found in DSD-0177 patient, who also harbored another *SRD5A2* variant p.R227Q. Synonymous variants are typically regarded as benign, yet they may exert pathogenic effects by affecting mRNA stability [25]. Further functional assays are needed to ascertain the pathogenicity of such variants. Notably, positions 6, 196, 203, 227, 235 and 246 are recognized hotspots within the *SRD5A2* gene [26, 27]. Among our patient cohort, *SRD5A2* R227Q was the most prevalent variant, followed by R246Q. In a previous study involving 190 individuals from diverse regions in China, the prevalence of R227Q in south China was found to be 62.62% [28]. In our cohort, approximately 64.7% (11/17) of patients carried R227Q, with 5 patients being homozygosity and 6 being compound heterozygosity. Nonetheless, no distinct genotype–phenotype correlations were observed among these patients. Interestingly, two patients (DSD-0249 and DSD-0278) diagnosed with micropenis carry only one heterozygous variant of *SRD5A2* gene, with no additional pathogenic variants identified, which cannot explain the phenotype. This is likely due to the panel’s incapacity to detect copy number variations (CNVs) or intronic region variants. Whole Exome Sequencing (WES) or Whole Genome Sequencing (WGS) would be valuable to uncover additional *SRD5A2* variants or the other causative genetic factors in these patients. DSD-0251 was provisionally diagnosed with 5α-reductase 2 deficiency based on the assessment of T/DHT level, but no *SRD5A2* variant was detected. And endocrinological tests are not always reliable for diagnosing 5α-reductase 2 deficiency because T/DHT ratios following hCG stimulation vary according to the age and the severity of the enzyme defects [29, 30]. The diagnostic sensitivity of T/ DHT ratio of 10 is around 78%, but the specificity is only 72% [31]. Moreover, not all patients with 5-α reductase 2 deficiency diagnosed by clinical and hormonal findings were found to carry pathogenic variants in *SRD5A2* [30, 32]. Compared to those with genetically diagnosed 5-α reductase 2 deficiency, the average age is much younger in undiagnosed patients, at 1.3 ± 2.8 years old [30], consistent with our patient who was at 4 month old at diagnosis. This highlights the essential role of genetic testing for definitive diagnosis, especially in infants with clinical-hormonal diagnosis.

*AR* variants have primarily been associated with AIS. The AR protein consists of four functional domains: N-terminal domain (NTD), DNA binding domain (DBD), the C-terminal ligand binding domain (LBD), and a hinge region (HR) linking LBD and DBD [33]. In our cohort, 7 variants including two novel variants and five reported variants were identified. Notably, p.V731M has been recurrently observed as a somatic variant in prostate cancer patients [34–36], suggesting a potential association with an increased risk of testicular germ cell tumor (TGCT) in AIS patients [37]. While G444A was previously identified in colorectal cancer patients as a germline variant [38], it was first detected in a DSD patient in our study. Additionally, M887V and H886Y, previously reported in AIS patients [39, 40], were detected in DSD-0077, presenting ambiguous genitalia, and DSD-0292, displaying hypospadias. However, there were no *AR* or other gene variants detected in our three patients diagnosed with AIS. Interestingly, most patients in our cohort carrying *AR* variants exhibited no additional signs of androgen insensitivity beyond hypospadias and micropenis. This suggests that *AR* variants may be related to milder phenotypes, consistent with findings from previous studies [41, 42]. Although R841H has been identified as a hotspot variant in a Chinese cohort with a frequency of 5.6% (3/54) [20], it was not detect in our cohort.

*CHD7* is associated with CHARGE syndrome and hypogonadotropic hypogonadism-5 with or without anosmia [43]. Loss-of-function pathogenic variants in the *CHD7* gene account for approximately 65–70% of CHARGE syndrome cases [44]. However, not all patients carrying with *CHD7* variants develop classical CHARGE syndrom phenotype. In a study of 40 patients with idiopathic hypogonadotropic hypogonadism (IHH), two adult patients with cryptorchidism, abnormal testicular, and/or abnormal penis were identified to carry *CHD7* variants [45]. Another patient with middle hypospadias and right cryptorchidism and without other malformations was also found to carry a missense variant of *CHD7*, although the correlation between the phenotype and the variant could not be established [46]. Patients carry *CHD7* variants may also present with microphallus, cryptorchidism, and hypospadias without other malformations [21]. There is also a missense variant in *CHD7* reported in a patient with pure 46,XY gonadal dysgenesis in clinvar (VCV001344532.1 ). In our study, we identified *CHD7* variants in 3 patients with the phenotype of micropenis, cryptorchidism, or gonadal dysgenesis, no further clinical phenoypes were recorded. All these reports suggested that patients with *CHD7* variants may also present atypical CHARGE syndrome. However, the variant p.G1684S has been widely associated with CHARGE syndrome [47–50] .The other two variants are novel truncating *CHD7* variants reporting as de novo inheritance, a form of which was frequently identified in CHARGE syndrome than the other atypical CHARGE features [51]. Further clinical evaluations are needed for these patients. Unfortunately, due to lost follow-up for these three patients, we could not further assess the clinical manifestations. Patients with IHH typically exhibit no symptoms before puberty and it is particularly difficult to evaluate the hypothalamic-pituitary‐gonadal (H‐P‐G) axis inchildren [21], making it easy for their condition to be overlooked during childhood [52, 53]. In our cohort, all patients with variants associated with IHH exhibited no other malformations. These patients may require regular follow-up in the future. Furthermore, genetic testing is helpful for monitoring the prospective puberty.

In our cohort, only three *MAP3K1* variants were identified and all were VUS, which is consistent with findings in other regions of China [20] and Korea [54]. However, this incidence is much lower than what has been reported in Caucasians, where the prevalence of *MAP3K1* gene variants ranges from 15 to 20% [55]. These disparities suggest the genetic heterogeneity in European and Asian populations. In recent years, *GATA4* variants have been identified in patients with congenital heart disease(CHD) with various complications. *GATA4* variants have also been found in patients with 46,XY DSD due to impaired testis formation with or without CHD [56]. In this cohort, we observed three patients carrying a heterozygous *GATA4* variant P163S. Among them, one exhibited symptoms of atrial septal defect in addition to DSD, while the others did not show any CHD- associated symptoms. The *GATA4* P163S variant has been previously reported and linked to tetralogy of Fallot [57]. The presence of multiple phenotypes among *GATA4* variants carriers may be attributed to incomplete penetrance, variable interactions with partner genes, and oligogenic mechanisms. Nonetheless, it is important to note that patients without CHD phenotypes also require diligent monitoring of their cardiac health in the future.

In this study, we also identified several relatively uncommon causative genes associated with DSD, including *TP63* and *GNAS*. Notably, *TP63* has not been previously reported in DSD patients, with only a limited number of studies indicating its association with ovarian development in *TP63* knockout mice [58, 59]. *GNAS* is a complex imprinted gene locus and encodes multiple transcripts sharing exons 2–13 but with alternative first exons [60]. Genetic defects affecting *GNAS* can cause several human diseases such as Pseudopseudo hypoparathyroidism (PHP, MIM:612462, 603233, 103580 ), end-organ resistance to parathyroid hormone (PTH, MIM: 612463), progressive osseous heteroplasia (POH, MIM: 166350). Somatic variants can also found in multiple benign and malignant tumors and McCune-Albright syndrome (MIM: 174800). The *GNAS* variant p.G142X we identified is located in the first exon of XLas (NM_080425.3) transcript, which is primarily expressed in neuroendocrine tissues and essential for normal fetal growth and development [61, 62]. This variant was initially reported in a patient with high bone mass, unclosed cranial suture, persistent hypophosphatemia, and elevated parathyroid hormone (PTH) levels [61]. However, genital development was not assessed in this family. The variant was inherited from the patient’s father and transmitted to his daughter. Both the proband and his father experienced bone fractures, while his 12-year-old daughter showed no symptoms. Both of our patients, DSD-0266 and DSD-0322, carry the same nonsense variant p.G142X. Recently, patient DSD-0266 presented with bone and joint instability and growth retardation (April,2024). He had elevated alkaline phosphatase (ALP) levels and an estradiol level of 124 pmol/L, but normal with thyroid function tests. Unfortunately, patient DSD-0322 was lost to follow-up. The cases of our patients, along with the previous reported cases, expand the spectrum of clinical manifestations associated with *GNAS* pathogenic variants. Interestingly, the absence of symptoms in the girl carrying this variant suggests the p.G142X variant in XLas may affect males exclusively. Further investigations will be conducted to explore the role of this variant.

In addition to monogenic variants, it is crucial to recognize the potential involvement of oligogenic variants in cases where patients exhibit negative results. Previous studies underscore that fewer than 50% of individuals with DSD receive a definitive genetic diagnosis, prompting the exploration of oligogenic diseases [56]. We have identified 9 patients with multiple variants with potential interaction and disease causing pathogenicity. These patients present with DSD phenotypes such as micropenis, cryptorchidism, hypospadias, or combination of these conditions. However, even when carrying the same genetic variant, the phenotype of each patient varies, suggesting other genetic modifiers may influence the unique phenotype of each patient. Furthermore, it is suggested that the greater the number of affected genes and variants an individual harbors, the more severe or variable the phenotype is likely to be [63, 64]. We found four patients had known pathogenic variants in *PROKR2* combined with other DSD gene variants and one patient with monoallelic *PROKR2* variant. Among them, three patients habored the most common *PROKR2* W178S variant in the Chinese CHH population [65]. Although all three present with micropenis, patient DSD-0115 with *ATRX* P1548L had right cryptorchidism, laterla hernia, and hyposmia, while DSD-0260 with both *ATRX* and *AR* variants didn’t show any other phenotypes in recorded. Interestingly, patient DSD-0110 had both heterozygous *PROKR2* W178S variant and known causative homozygous *SRD5A2* p.R227Q variant, but no interaction was found in these two variants and the patient presented micropenis phenotype only. In contrast, the patient DSD-0016 with a monoallelic *PROKR2* variant presented with more severe phenotypes, including bilateral cryptorchidism, growth retardation, and facial dysmorphia. This is likely due to undiscovered genetic variants outside the panels we tested, which have pleiotropic roles that interact with the monoallelic *PROKR2* variant. The correlation between digenicity and severe phenotype was not always consistent [66]. This aligns with findings indicating that members of digenic families often had milder or similar phenotypes compared to probands carrying variants in the *FGFR1* gene and other genetic variants [66]. Our patients did not exhibit significant phenotypic differences whether they carried monoallelic *FGFR1* variants alone or in combination with other heterozygous variants in both autosomal dominant and recessive genes. This observation was also noted in patients carrying *NR5A1* variants alone or combined with other genetic variants. Nevertheless, the genotype-phenotype correlation in 46,XY DSD may largely depend on the nature of the secondary hit, explaining the broad spectrum of phenotypes [67]. However, we couldn’t establish a phenotype-genotype correlation, several factors may contribute to this, including the limited sample size with diverse genetic combinations hindering correlation efforts, and the absence of comprehensive clinical descriptions for all patients.

### Limitation

In our study, we observed a diagnostic rate of approximately 11.2%, and the rate is higher in trio/due patients (13.4%) than in singletons (8.6%). This rate is much lower than that observed in other studies conducted on the Chinese populations or other populations, where diagnostic rate exceeded 40%, especially in cohort using WES [19, 20, 68, 69]. Despite utilizing two panels with slightly different numbers of genes, our findings were limited by the absence of the latest DSD-related genes in the panels’ lists. WES which currently is the most effective and cost-efficient methods for identifying novel causes in undiagnosed DSD patients is the preferable tool. Less stringent patients’ selection criteria and the lack of clear differentiation of phenotype in patients may also limit our findings. Most of other groups are focusing on specific and severe phenotypes of 46,XY DSD such as 5α-reductase 2 deficiency, GD, AIS, HH[28, 70–72]. A multidisciplinary team and longitudinal follow-up should be conducted in recording physical examinations and biochemical (hormonal) evaluations. Combing these with genetic testing will improve the accuracy of genetic diagnoses. Additionally, it’s worth noting that we did not detect DNA copy number variations which may also contribute the phenotypes of DSD [73] due to the limitations of our gene panel. Furthermore, due to the lack of further validation, patients identified with VUS could not be conclusively diagnosed especially in singletons.

## Conclusion

In summary, we utilized the targeted gene panels to identify potential pathogenic gene variants in a large cohort of Chinese 46,XY DSD patients. This study represents the general investigation of such a large cohort of 46,XY DSD in eastern China, contributing to our understanding of the gene spectrum, mutation spectrum, and phenotypic spectrum of 46,XY DSD in this region. However, it’s important to acknowledge potential limitations in our study, particularly since all patients were from a single center and the gene panels used were relatively outdated. Therefore, future research should involve multi-center collaboration to further advance our knowledge in this field.

## Electronic supplementary material

Below is the link to the electronic supplementary material.

Figure S1: Sequencing quality control. (A) Fraction of target covered; (B) Average sequencing depth on target

Table S1 The detailed characteristics of each participant

## Data Availability

No datasets were generated or analysed during the current study.
