# Supplementary figures and images for: Application and insights of targeted next-generation sequencing in a large cohort of 46,XY disorders of sex development in Chinese
Source: Biol Sex Differ. 2024 Sep 16;15:73. doi: 10.1186/s13293-024-00648-6 (PMC11403886; doi:10.1186/s13293-024-00648-6)

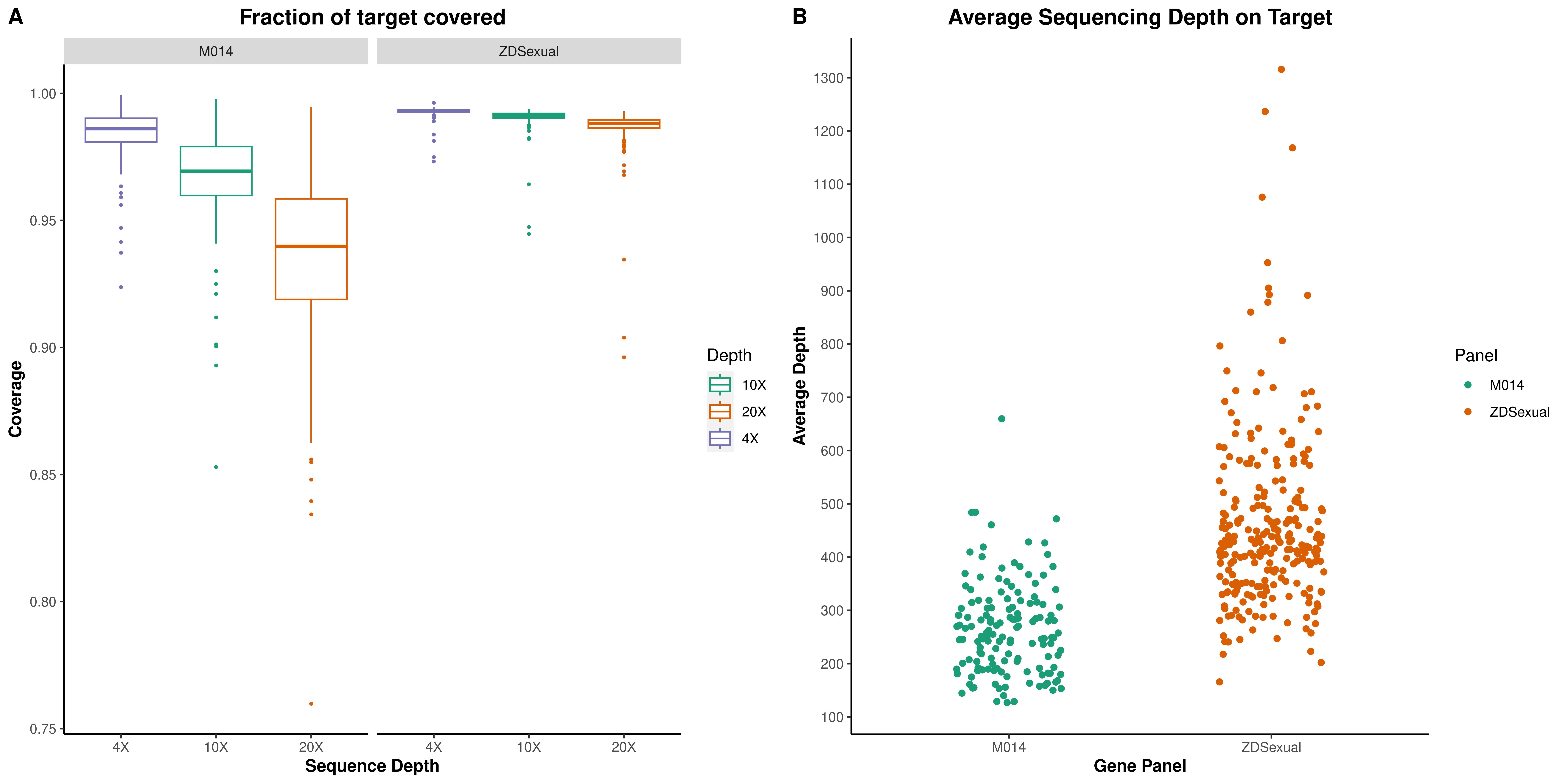

Supplement: Supplementary file 1 — Figure S1: Sequencing quality control. (A) Fraction of target covered; (B) Average sequencing depth on target [file 13293_2024_648_MOESM1_ESM.jpg]
